# Wall-Eyed Bilateral Internuclear Ophthalmoplegia as an Early Presentation of Multiple Sclerosis

**DOI:** 10.7759/cureus.36835

**Published:** 2023-03-28

**Authors:** Nuratiqah Zainal Abidin, Tengku Norina Tuan Jaffar, Liza Sharmini Ahmad Tajudin

**Affiliations:** 1 Department of Ophthalmology and Visual Sciences, School of Medical Science, Health Campus, Universiti Sains Malaysia, Kubang Kerian, MYS; 2 Ophthalmology, Raja Perempuan Zainab II Hospital, Kubang Kerian, MYS; 3 Ophthalmology Clinic, Hospital Universiti Sains Malaysia, Kubang Kerian, MYS

**Keywords:** binocular diplopia, multiple sclerosis, demyelinating disease, bilateral internuclear ophthalmoplegia, webino

## Abstract

Wall-eyed bilateral internuclear ophthalmoplegia (WEBINO) is a rare neuro-ophthalmological condition in which there is ocular motility impairment characterized by bilateral adduction deficiencies, bilateral abducting nystagmus, and exotropia in primary gaze, and is often associated with multiple sclerosis (MS). This report describes a young female who presented with sudden onset of binocular diplopia and alternating exotropia for two days duration, which was associated with a history of intermittent headaches for one year before presenting complaints. Examination revealed alternating exotropia on the primary gaze with bilateral limitation of adduction and bilateral nystagmus on abduction. Other ocular and neurological examinations were unremarkable. Neuroimaging showed multiple white matter lesions that were consistent with demyelinating disease. Her symptoms completely resolved after the initiation of intravenous corticosteroid therapy. However, she developed left upper limb numbness four months later, and a repeat magnetic resonance imaging (MRI) of the brain showed the presence of multiple new brain lesions. Subsequently, she was diagnosed with MS and started on immunotherapy. Her symptoms resolved, with no residual ophthalmoplegia or any neurological symptoms.

## Introduction

Multiple sclerosis (MS) is an autoimmune demyelinating nervous system disease that often involves the visual system and may present first with ophthalmologic symptoms [1]. The most common ocular manifestation of MS is optic neuritis, however, ocular motor deficits such as internuclear ophthalmoplegia (INO), either unilateral or bilateral, are also being reported in MS patients [2, 3]. Wall-eyed bilateral internuclear ophthalmoplegia (WEBINO) is an adduction deficit of bilateral eyes with exotropia on primary gaze, and the major lesion in this condition is thought to be in the bilateral medial longitudinal fasciculi (MLF) [4]. We present the case of a female who presented initially with WEBINO and subsequently was diagnosed with MS.

## Case presentation

A 29-year-old female who was premorbidly healthy reported with sudden onset of binocular diplopia and alternating exotropia two days before presentation. She had an intermittent headache for the past year which improved with analgesics. Otherwise, she had no limb weakness, increased intracranial pressure symptoms, tinnitus, or constitutional symptoms. She was not taking any contraceptive pills or steroid supplements. She was medium-built and was not obese.

**Figure 1:**
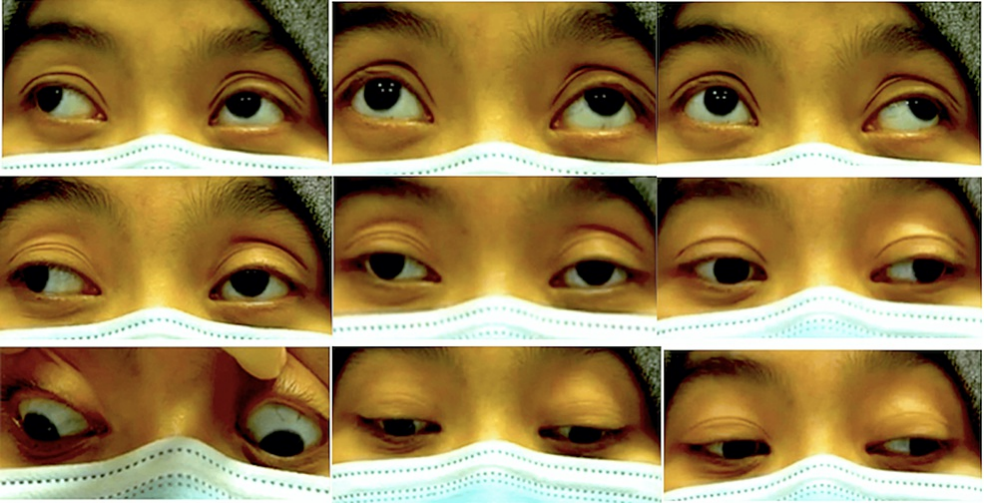
Showing nine cardinal gazes of the patient at the initial presentation. On primary gaze, the left eye deviates outward. There are bilateral adduction deficits on horizontal gaze and nystagmus of abducting eyes, respectively.

Upon presentation, her visual acuity for both eyes was 6/9. There was no relative afferent pupillary defect. On primary gaze, there was alternating exotropia bilaterally. The right eye was deviating outward on the fixation with the left eye, while the left eye was deviating outward on the fixation with the right eye. Bilateral adduction deficits were noted on horizontal gaze, together with nystagmus of abducting eyes respectively, and diplopia at all gazes (Figure 1). Convergence was impaired, but the vertical gaze was preserved. Otherwise, there was no abnormal head posture, and no proptosis or ptosis was noted. Anterior segment and posterior segment examinations of both eyes were unremarkable, with no signs of papilloedema seen. Her neurological examinations, including cerebellar signs and cranial nerve examinations, were also normal.

**Figure 2:**
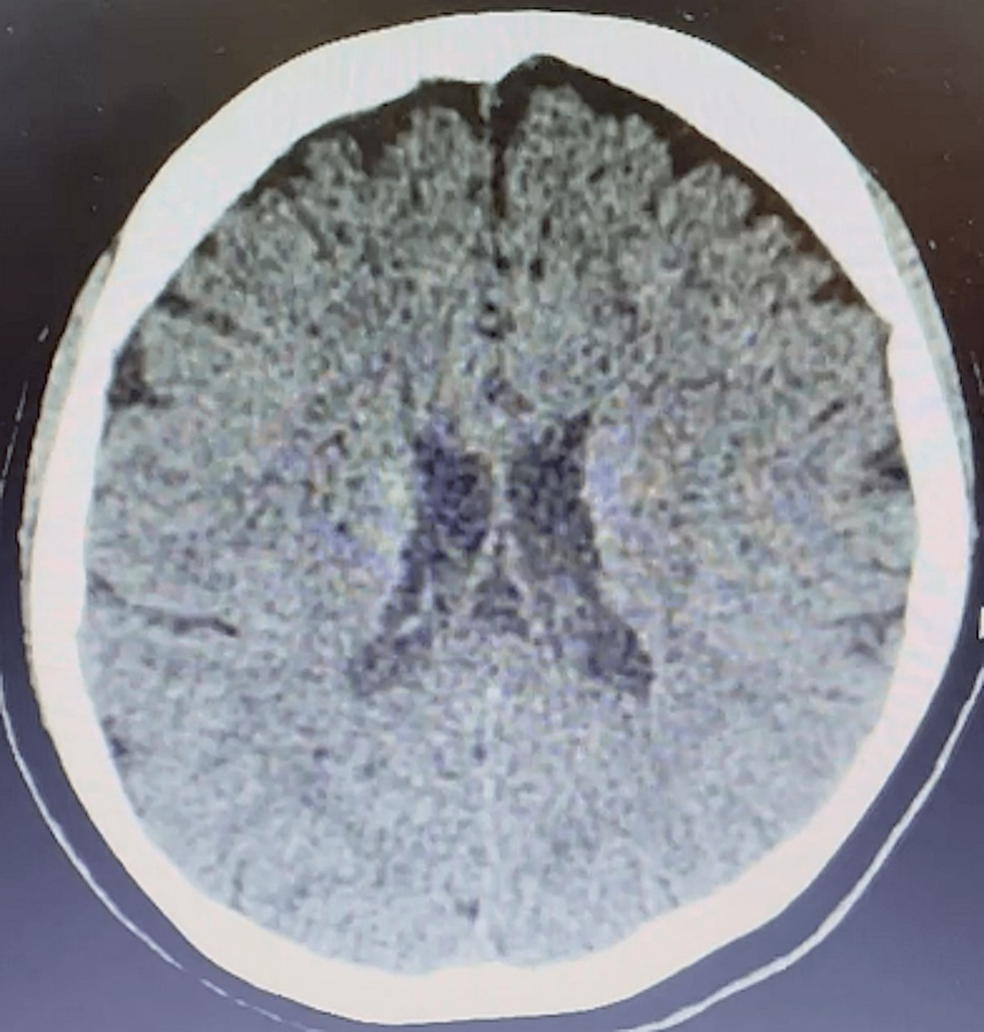
Contrasted computed tomography imaging of the brain revealed normal findings

**Figure 3:**
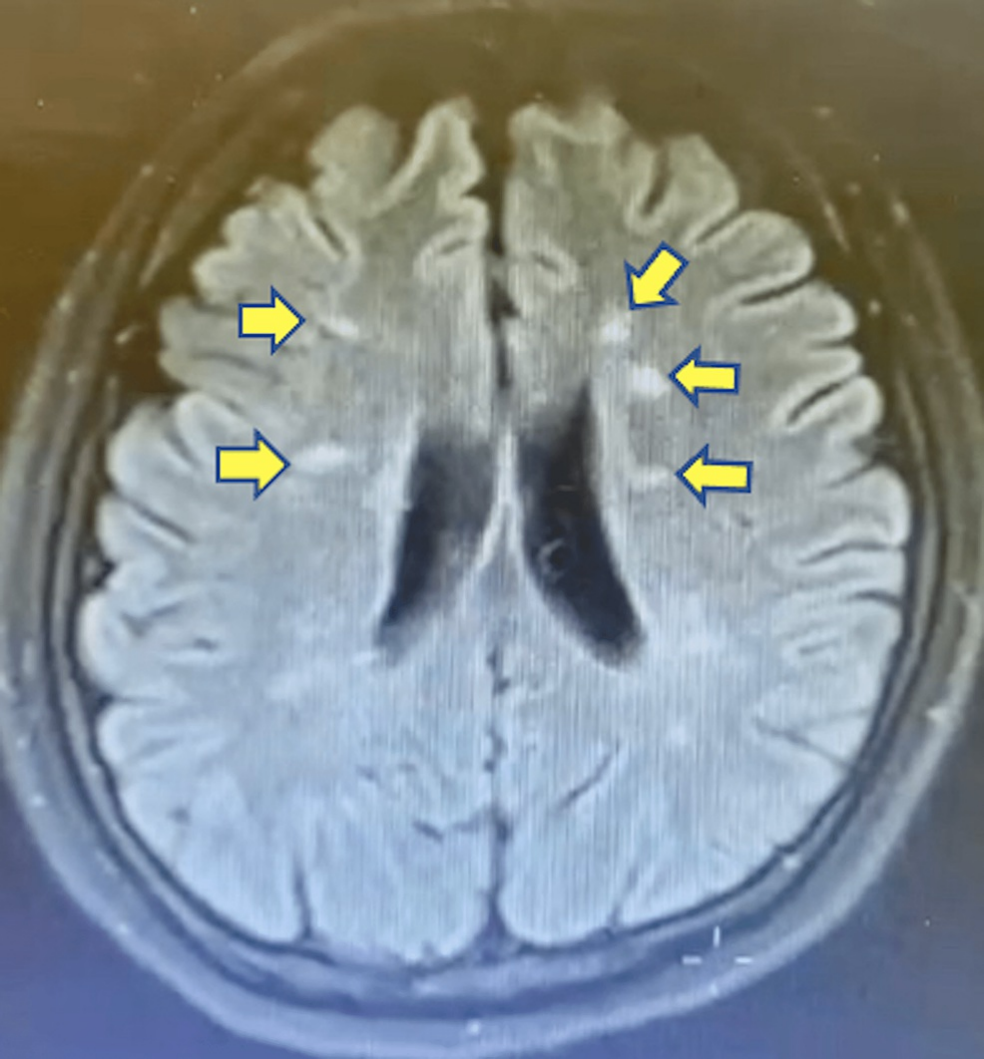
Axial view of an MRI FLAIR of the brain showing multiple periventricular white matter lesions (arrow) with Dawson's fingers appearance FLAIR: Fluid-attenuated inversion recovery

**Figure 4:**
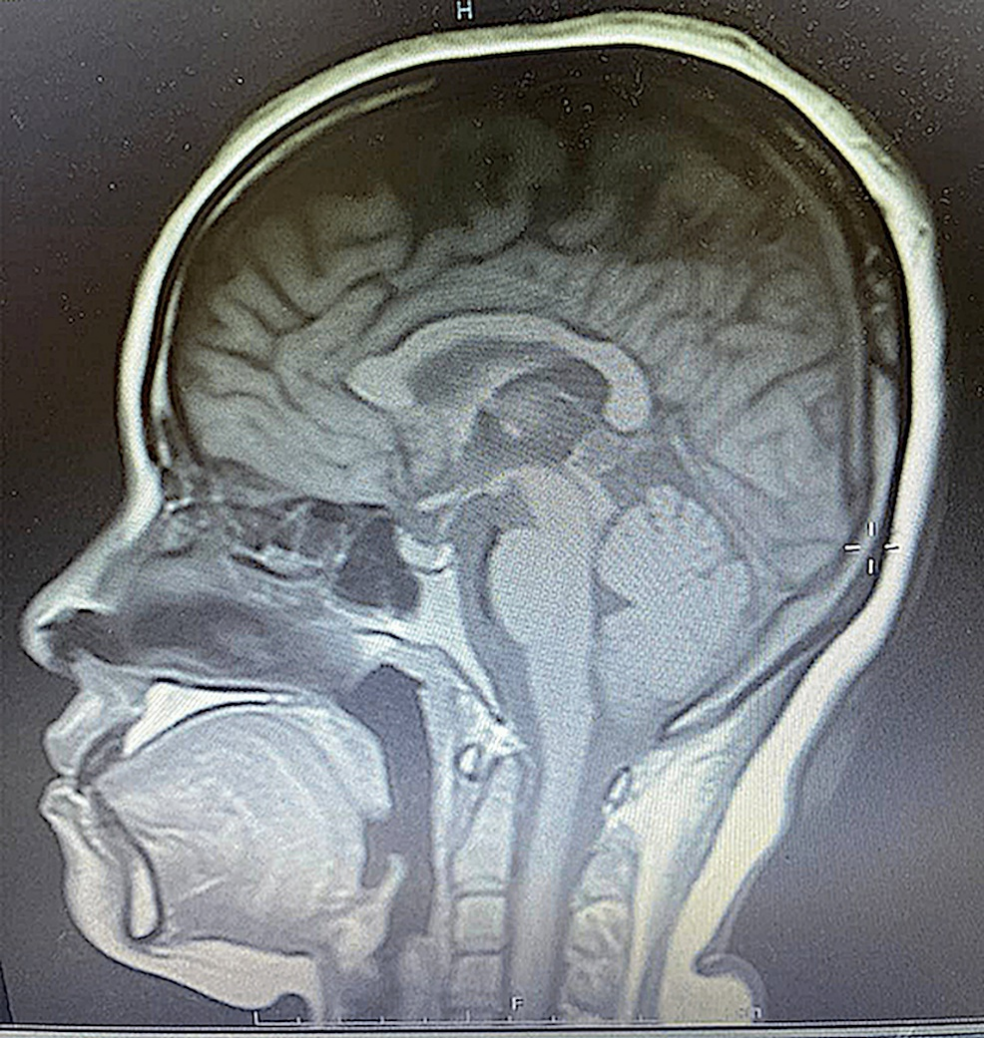
Sagittal view of the brainstem on MRI showing normal findings

Contrasted computed tomography imaging of the brain showed no abnormalities (Figure 2). However, her MRI of the brain revealed the presence of multiple hyperintense white matter lesions in various areas of the brain, including the cerebrum, corpus callosum, the left cerebellar peduncle, and the periventricular region, which is indicative of demyelinating disease and known as "Dawson's fingers" (Figure 3). However, the brainstem showed normal results on the MRI (Figure 4).

Subsequently, she was referred to the neuromedical team for further management. Lumbar puncture revealed normal opening pressure with no evidence of infection. Oligoclonal bands, aquaporin-4 receptor antibody, and myelin oligodendrocyte glycoprotein (MOG) antibody were negative. She was started on intravenous methylprednisolone 500 mg daily for five days and was discharged with oral prednisolone for 11 days. However, no maintenance therapy or immunomodulatory therapy was administered to her at that time. Her symptoms completely resolved after two weeks of being discharged from the hospital with no residual ophthalmoplegia, and her neurological examinations were also unremarkable. An MRI of the whole spine, which was done two months later, also showed no evidence of focal spinal cord lesions.

Four months later, she developed left upper limb numbness for three weeks, which was not improved with medications. However, there was no limb weakness or any new ocular symptoms. Examination revealed an unremarkable neurological assessment except for reduced sensory over the left upper limb. Repeated MRI of the brain showed the presence of multiple new lesions seen at both periventricular regions, cerebrum, and occipital areas that were consistent with McDonald criteria, which are dissemination in space and time. Subsequently, the patient was diagnosed with relapse remitting multiple sclerosis (RRMS) and was started on subcutaneous interferon injection, and her symptoms improved with no residual ophthalmoplegia or any neurological symptoms.

## Discussion

This report presents the case of a 29-year-old female who developed bilateral INO with convergence impairment, bilateral abducting nystagmus, and exotropia in the primary gaze. INO is characterized by abnormal horizontal ocular movement, including lost or limited adduction in the ipsilateral eye and horizontal abducting nystagmus in the contralateral eye [1, 2, 4]. This occurs as a result of lesions involving the MLF in the midbrain, which carries an adduction signal during horizontal movement from the contralateral abducens nucleus to the ipsilateral oculomotor nucleus [1, 2, 4].

When a lesion occurs in the MLF on both sides, it affects the transmission of signals for horizontal conjugate eye movement from the abducens nucleus to the contralateral medial rectus subnucleus of the oculomotor nerve [3, 4]. This disruption of connections between the abducens nucleus and the contralateral medial rectus subnucleus leads to bilateral adduction deficits. Nevertheless, the innervation to each eye's lateral rectus on the same side as the lesion remains unaffected. The exotropia observed in WEBINO has been theorized to be caused by a lesion in the pontine section of the brainstem or by direct involvement of the oculomotor nerve nucleus located in the midbrain [4].

The cause of abducting nystagmus can be explained by Hering’s law of equal innervation, which states that yoked extraocular muscles receive an equal degree of innervation [3, 4]. Due to the paresis, the weakened medial rectus muscle triggers the higher oculomotor control centers to enhance innervation to the medial rectus as a compensatory mechanism. With the paretic medial rectus receiving extra innervation, the contralateral lateral rectus, which is its corresponding synergist muscle, also gets additional innervation. This leads to an excessive innervation in the contralateral lateral rectus, causing nystagmus when the contralateral eye overcompensates and then self-corrects for the overshoot of the target [4].

Unilateral or bilateral INO has been associated with several systemic disorders, including MS, with prevalence estimates ranging from 17% to 41% [5]. Bilateral INO could be seen with a single brainstem lesion or multiple lesions affecting the MLFs on both sides, and the causes can be due to ischemic, autoimmune conditions such as MS, infectious, inflammatory, toxic, nutritional, traumatic, or metabolic [1, 6, 7]. Even though INO is the most common saccadic disorder seen in MS, it is uncommon for individuals to present with bilateral INO in their initial clinical presentation, such as in our case report. The majority of case reports or case series on MS-related WEBINO have originated from countries such as the United States of America, the United Kingdom, Japan, and Turkey. However, there have been no reported instances of bilateral INO resulting from MS in Malaysia, possibly because the presentation is uncommon or has not been previously reported. Therefore, our case represents the first documented instance of bilateral INO resulting from MS in Malaysia.

The most common ocular symptom in MS is optic neuritis, which can occur in up to 50% of patients and is the disease's initial clinical presentation in 20% of individuals [1, 3]. However, patients with MS may experience a range of ocular motor impairments such as unilateral or bilateral INO. INO in MS can manifest as an acute symptom of an MS attack or a chronic problem due to incomplete recovery from a previous attack or the chronic phase of the disease [1, 5].

Lesions in MLF are generally very small and may or may not be visible on MRI [1]. The T2-weighted MRI sequences are more effective than fluid-attenuated inversion recovery (FLAIR) images at identifying MLF participation [1]. In our case report, the initial and repeated MRI of the brain shows multiple hyperintense white matter lesions in both cerebri, corpus callosum, left cerebellar peduncle, periventricular region, and occipital areas that are consistent with the McDonald criteria of MS which are dissemination in space (DIS) and dissemination in time (DIT) [8], however, similar lesions in the MLF are not visible in both studies. This is probably due to the very small lesion in MLF that was not detected with neuroimaging.

Our case report describes a young woman who experienced two acute attacks of MS within a year, despite having no family history of the disease. Both her initial and repeated brain MRI scans were consistent with the McDonald criteria for DIT and DIS in 2010, and as a result, she began receiving subcutaneous interferon injections after the second attack. After one year of follow-up, her symptoms improved, with no residual ophthalmoplegia or neurological deficits.

## Conclusions

Ocular symptoms may be the first sign of MS. Bilateral INO may indicate a significant chance of developing MS in patients without a known systemic demyelinating condition, especially in a young patient. Recognizing ocular signs and prompting neuroimaging help clinicians properly evaluate the differential diagnosis and effective treatments for MS.
